# Supplementary material for: Novel Cytonuclear Combinations Modify Arabidopsis thaliana Seed Physiology and Vigor
Source: Front Plant Sci. 2019 Feb 5;10:32. doi: 10.3389/fpls.2019.00032 (PMC6370702; doi:10.3389/fpls.2019.00032)
Supplement: Supplementary file 8 [file Presentation_1.pdf]

Methods S1 Statistical models and contrast tests

In the following, the model selected for each experiment is described and the contrast tests defined:

Data from seed dormancy assays were analyzed independently at each post-harvesting date. The [Sha]Cvi-0 and [Ct-1]Jea genotypes were not included in the analysis due to missing data. All genotypes with the Ita-0 nucleus were also excluded due to no or very low germination in any conditions. Except for 0PH where only seed data at 15°C were analyzed, in all models, considered factors were shelf (position of the plant in the growth chamber), harvest (biological repetition, *i.e.* independent production), temperature (in the germination test), cytoplasm and nucleus.

- For 0PH, genotypes [Blh-1]Cvi_0, [Jea]Cvi-0 and [Oy-0]Cvi-0 were excluded from the analysis because of total absence of germination. The selected model was the one with all the first and second order terms plus 2 third order interactions shelf x harvest x cytoplasm and shelf x harvest x nucleus.

Effect of each foreign cytoplasm (c) in each natural accession nuclear background (c', n') was tested with the hypothesis

H0 $\{(C_c - C_{c'}) + (CN_{cn'} - CN_{c'n'})$

$+ 1/2\Sigma_{h \text{ in } \{1,2\}} (HC_{hc} - HC_{hc'}) + 1/2\Sigma_{s \text{ in } \{1,2\}} (SC_{sc} - SC_{sc'})$

$+ 1/4\Sigma_{h \text{ in } \{1,2\}}\Sigma_{s \text{ in } \{1,2\}} (HSC_{hsc} - HSC_{hsc'}) = 0\}$

Hence the cytoplasm effects in each nuclear background should be interpreted as averaged on the harvests and the shelves.

An effect of the cytonuclear interacting combination was tested for all pairs of cytoplasms (c, c') and for all pairs of nuclei (n, n'). As no third order interaction involving both nucleus and cytoplasm was included in the selected model, cytonuclear interacting combinations could be tested independently from other model terms. Each was tested with the hypothesis

H0 $\{(CN_{cn} - CN_{c'n}) - (CN_{cn'} - CN_{c'n'}) = 0\}$,

where CN states for the model term corresponding to the second-order interaction between cytoplasm and nucleus.

- For 3PH, the selected model was the one with all the first and second order terms plus 4 third

order interactions shelf x cytoplasm x nucleus (SCN), harvest x cytoplasm x nucleus (HCN), shelf x harvest x nucleus and harvest x temperature x nucleus.

Effect of each foreign cytoplasm (c) in each natural accession nuclear background (c', n') was tested with the hypothesis

$$H0 \{(C_c - C_{c'}) + (CN_{cn'} - CN_{c'n'}) + 1/2\Sigma_{t \text{ in } \{1,2\}} (TC_{tc} - TC_{tc'})$$

$$+ 1/2\Sigma_{h \text{ in } \{1,2\}} (HC_{hc} - HC_{hc'}) + 1/2\Sigma_{h \text{ in } \{1,2\}} (HCN_{hcn'} - HC_{hc'n'})$$

$$+ 1/2\Sigma_{s \text{ in } \{1,2\}} (SC_{sc} - SC_{sc'}) + 1/2\Sigma_{S \text{ in } \{1,2\}} (SCN_{hcn'} - SC_{hc'n'}) = 0\}$$

Hence the cytoplasm effects in each nuclear background should be interpreted as averaged on the germination temperatures, the harvests and the shelves.

An effect of the cytonuclear interacting combination was tested for all pairs of cytoplasms (c, c') and for all pairs of nucleus (n, n'). As the selected model contained only the third order interaction terms involving cytoplasm and nucleus SCN and HCN, cytonuclear interacting combinations could be tested independently from temperature. Each was tested with the hypothesis

$$H0 \{ [(CN_{cn} - CN_{c'n}) - (CN_{cn'} - CN_{cn'})$$

$$+ 1/2\Sigma_{h \text{ in } \{1,2\}} [(HCN_{hcn} - HCN_{hc'n}) - (HCN_{hcn'} - HCN_{hcn'})]$$

$$+ 1/2\Sigma_{s \text{ in } \{1,2\}} [(SCN_{scn} - SCN_{sc'n}) - (SCN_{scn'} - SCN_{sc'n'})]] = 0\}.$$

Hence the cytonuclear interacting combination effects should be interpreted as averaged on the harvests and the shelves.

- For 6PH, the selected model was the one with all the first and second order terms plus 4 third order interactions harvest x cytoplasm x nucleus (HCN), shelf x harvest x nucleus, shelf x temperature x nucleus and shelf x harvest x temperature.

Effect of each foreign cytoplasm (c) in each natural accession nuclear background (c', n') was tested with the hypothesis

$$H0 \{(C_c - C_{c'}) + (CN_{cn'} - CN_{c'n'}) + 1/2\Sigma_{t \text{ in } \{1,2\}} (TC_{tc} - TC_{tc'})$$

$$+ 1/2\Sigma_{h \text{ in } \{1,2\}} (HC_{hc} - HC_{hc'}) + 1/2\Sigma_{s \text{ in } \{1,2\}} (SC_{sc} - SC_{sc'})$$

$$+ 1/2\Sigma_{h \text{ in } \{1,2\}} (HCN_{scn'} - HCN_{hc'n'}) = 0\}$$

Hence the cytoplasm effects in each nuclear background should be interpreted as averaged on the temperatures and the harvests.

An effect of the cytonuclear interacting combination was tested for all pairs of cytoplasms (c,

c') and for all pairs of nucleus (n, n'). As the selected model contained only the third order interaction term involving cytoplasm and nucleus HCN, cytonuclear interacting combinations could be tested independently from germination temperature and shelf. Each was tested with the hypothesis

$H0 \{[(CN_{cn} – CN_{c'n}) – (CN_{cn'} – CN_{cn'})$

$+ 1/2\Sigma_{h \text{ in } \{1,2\}} [(HCN_{hcn} – HCN_{hc'n}) – (HCN_{hcn'} – HCN_{hcn'})] = 0\}$.

Hence the cytonuclear interacting combination effects should be interpreted as averaged on the harvests.

- For 9PH, the selected model was the one with all the first and second order terms plus 4 third order interactions harvest x cytoplasm x nucleus (HCN), shelf x harvest x nucleus, harvest x temperature x nucleus and shelf x harvest x temperature.

Effect of each foreign cytoplasm (c) in each natural accession nuclear background (c', n') was tested with the hypothesis

$H0 \{(C_c – C_{c'}) +(CN_{cn'} – CN_{c'n'}) + 1/2\Sigma_{t \text{ in } \{1,2\}} (TC_{tc} – TC_{tc'})$

$+ 1/2\Sigma_{h \text{ in } \{1,2\}} (HC_{hc} – HC_{hc'}) + 1/2\Sigma_{s \text{ in } \{1,2\}} (SC_{sc} – SC_{sc'})$

$+ 1/2\Sigma_{h \text{ in } \{1,2\}} (HCN_{scn'} – HCN_{hc'n'}) = 0\}$

Hence the cytoplasm effects in each nuclear background should be interpreted as averaged on the temperatures and the harvests.

An effect of the cytonuclear interacting combination was tested for all pairs of cytoplasms (c, c') and for all pairs of nucleus (n, n'). As the selected model contained only the third order interaction term involving cytoplasm and nucleus HCN, cytonuclear interacting combinations could be tested independently from germination temperature and shelf. Each was tested with the hypothesis

$H0 \{[(CN_{cn} – CN_{c'n}) – (CN_{cn'} – CN_{cn'})$

$+ 1/2\Sigma_{h \text{ in } \{1,2\}} [(HCN_{hcn} – HCN_{hc'n}) – (HCN_{hcn'} – HCN_{hcn'})] = 0\}$.

Hence the cytonuclear interacting combination effects should be interpreted as averaged on the harvests.

<u>Data from germination performance after storage</u>.

As the salt concentration was adjusted to the tolerance of the nuclear background of the lines,

and in order to allow testing of cytonuclear interacting combinations, the data were analyzed considering the salt term was encoded as a binary factor (presence or absence).

The selected model was the one with the first order terms salt, cytoplasm and nucleus plus all second-order interactions, namely cytoplasm x nucleus, cytoplasm x salt and nucleus x salt. As the third-order interaction cytoplasm x nucleus x salt was not included in the selected model, cytonuclear interacting combinations could be tested independently from the presence of salt.

Effect of each foreign cytoplasm (c) in each natural accession nuclear background (c', n') was tested with the hypothesis

H0 $\{(C_c - C_{c'}) + (CN_{cn'} - CN_{c'n'}) + 1/2\Sigma_{s \text{ in } \{0,1\}} (SC_{sc} - SC_{sc'}) = 0\}$, where CN states for the model term corresponding to the second-order interaction between cytoplasm and nucleus and SC for the model term corresponding to the second-order interaction between salt and cytoplasm. Hence the cytoplasm effects in each nuclear background should be interpreted as averaged on the presence/absence of salt.

An effect of the cytonuclear interacting combination was tested for all pairs of cytoplasms (c, c') and for all pairs of nucleus (n, n') with the hypothesis

H0 $\{(CN_{cn} - CN_{c'n}) - (CN_{cn'} - CN_{c'n'}) = 0\}$

<u>Data from seed longevity</u> in the Sha and Ct-1 nuclear backgrounds.

The results were analyzed separately for the two nuclear backgrounds.

For some levels of the aging treatment time length (aatime = 0 and 40) for Ct-1nuclear background, the percentage of germination was exactly equal to 0 or 100%. This led to an unstable estimation of the model parameters due to the data being linearly separable. Consequently, the data corresponding to these aatime levels were discarded in the analyses for the two nuclear backgrounds.

In each nuclear background, the selected model was the complete model: cytoplasm (C) + aatime (A) + cytoplasm x aatime (CA). The cytoplasm effect was tested for all pairs of cytoplasms (c, c') with the hypothesis

H0 $\{[(C_c - C_{c'}) + 1/3\Sigma_{a \text{ in } \{10,20,30\}} (CA_{ca} - CA_{c'a})] = 0\}$

Hence the cytoplasm effect should be interpreted as averaged on the three aatimes.

<u>Germination with exogenous ABA</u>

Seed germination was analyzed as previously described (see main text material and methods). The selected model was the complete model: cytoplasm (C) + treatment (T) + cytoplasm x

treatment (CT). Because we were interested in sensitivity to ABA, we tested the interaction term CT for the three cytoplasm pairs (c,c'), and the treatment pair (a,w, for ABA and water), with the hypothesis

H0 $\{(CT_{ca} - CT_{c'a}) - (CT_{cw} - CT_{c'w}) = 0\}$

Dosages of ABA content

The data from dry seeds, seeds germinated on water and seeds germinated on salt were analyzed independently. In each case the data from the two independent experiments were jointly analyzed.

For dry seeds and seeds germinated on water, the selected models did not contain the cytoplasm x experiment interaction term, so in these two cases, cytoplasm effects for the three cytoplasm pairs (c, c') could be tested independently from the experiment with the hypothesis

H0 $\{(C_c - C_{c'}) = 0\}$

For seeds germinated on salt, the selected model contained the interaction term cytoplasm x experiment. So, the cytoplasm effect was tested for the three pairs of cytoplasms (c, c') with the hypothesis

H0 $\{[(C_c - C_{c'}) + 1/2\sum_{e \text{ in } \{1,2\}} (CE_{ce} - CE_{c'e})]=0\}$

Hence in this case, the cytoplasm effect should be interpreted as averaged on the two experiments.

Dosages of ion content

The ratios Na^+/K^+ were analyzed only for NaCl treated seeds, independently at T0 (just after stratification) and at T20 (after 20 hours in germination conditions). The linear models thus only contained the cytoplasm term and the cytoplasm effects were tested for the three pairs of cytoplasms (c, c') with the hypothesis

H0 $\{(C_c - C_{c'}) = 0\}$
