# Supplementary material for: Novel Cytonuclear Combinations Modify Arabidopsis thaliana Seed Physiology and Vigor
Source: Front Plant Sci. 2019 Feb 5;10:32. doi: 10.3389/fpls.2019.00032 (PMC6370702; doi:10.3389/fpls.2019.00032)
Supplement: Supplementary file 9 [file Data_Sheet_1.pdf]

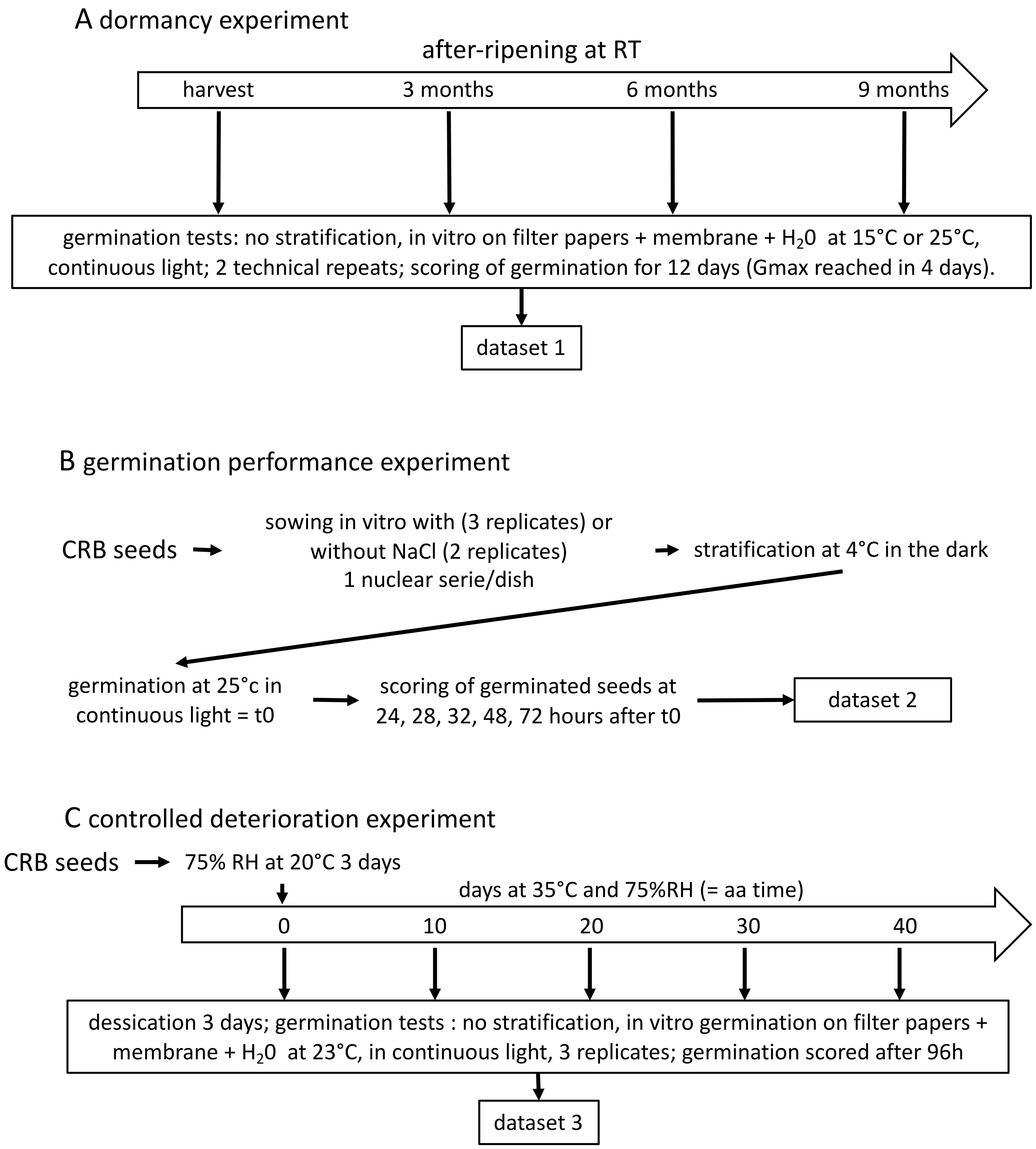

Fig S1 Schematic of experimental procedures for the production of datasets

datasets 1, 2 and 3 are provided in Supplementary Tables S3, S5 and S6, respectively
