# Supplementary material for: Novel Cytonuclear Combinations Modify Arabidopsis thaliana Seed Physiology and Vigor
Source: Front Plant Sci. 2019 Feb 5;10:32. doi: 10.3389/fpls.2019.00032 (PMC6370702; doi:10.3389/fpls.2019.00032)
Supplement: Supplementary file 10 [file Data_Sheet_2.PDF]

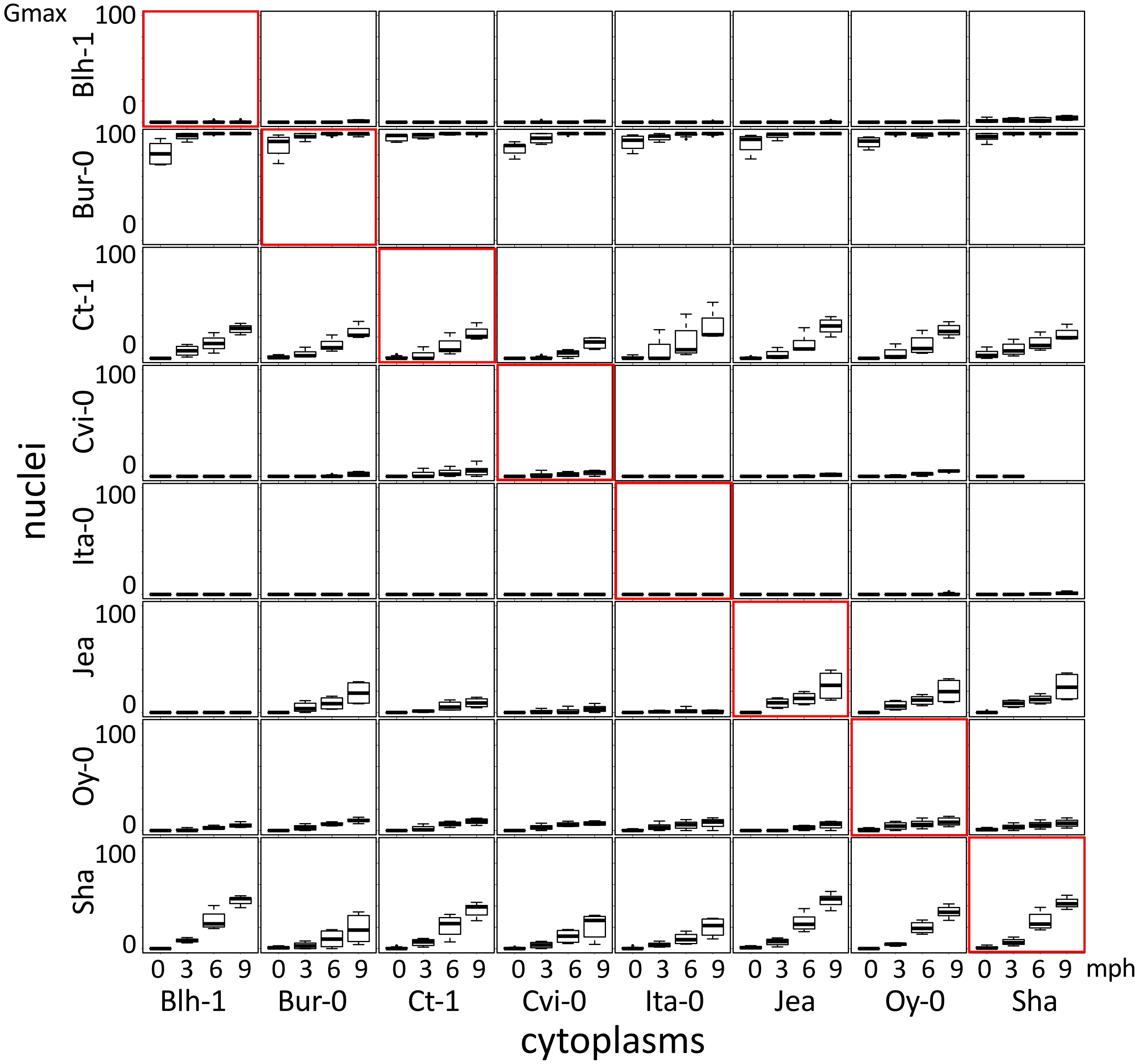

Fig. S2 Dormancy release during after ripening in cytoline series.

Each panel shows the maximum germination percentage at 25°C for one cytonuclear combination 0, 3, 6 and 9 months post-harvest (mph). Rows present cytolines sharing their nucleus and columns those sharing their cytoplasm. Natural accessions are framed in red.
