# Supplementary material for: Novel Cytonuclear Combinations Modify Arabidopsis thaliana Seed Physiology and Vigor
Source: Front Plant Sci. 2019 Feb 5;10:32. doi: 10.3389/fpls.2019.00032 (PMC6370702; doi:10.3389/fpls.2019.00032)
Supplement: Supplementary file 11 [file Data_Sheet_3.PDF]

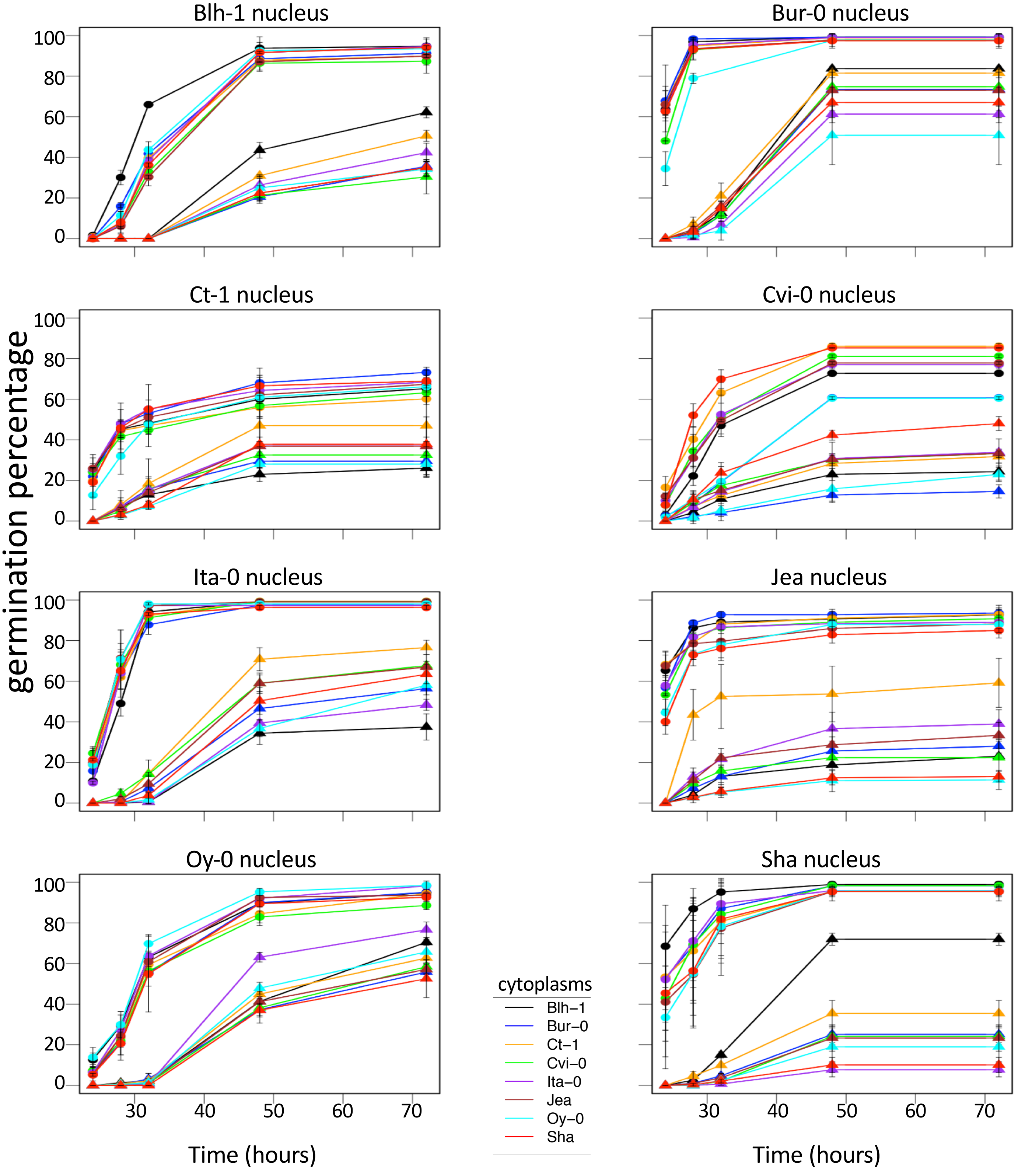

Fig. S3 Germination time curves of cytoline series on water and NaCl.

Each panel represents the germination kinetics of cytolines sharing the same nucleus, either on water (dots) or on salt (triangles). The colors of the graphs indicate the cytoplasms of the genotypes: black, Blh-1; blue, Bur-0, orange, Ct-1; green, Cvi-0; purple, Ita-0; brown, Jea; cyan, Oy-0; red, Sha. Error bars indicate the SD around the plotted mean for germination, from two replicates on water, and three replicates on salt. The NaCl concentration used was chosen after preliminary experiments on the corresponding natural accession (Table S1).
