# Supplementary material for: Novel Cytonuclear Combinations Modify Arabidopsis thaliana Seed Physiology and Vigor
Source: Front Plant Sci. 2019 Feb 5;10:32. doi: 10.3389/fpls.2019.00032 (PMC6370702; doi:10.3389/fpls.2019.00032)
Supplement: Supplementary file 12 [file Data_Sheet_4.PDF]

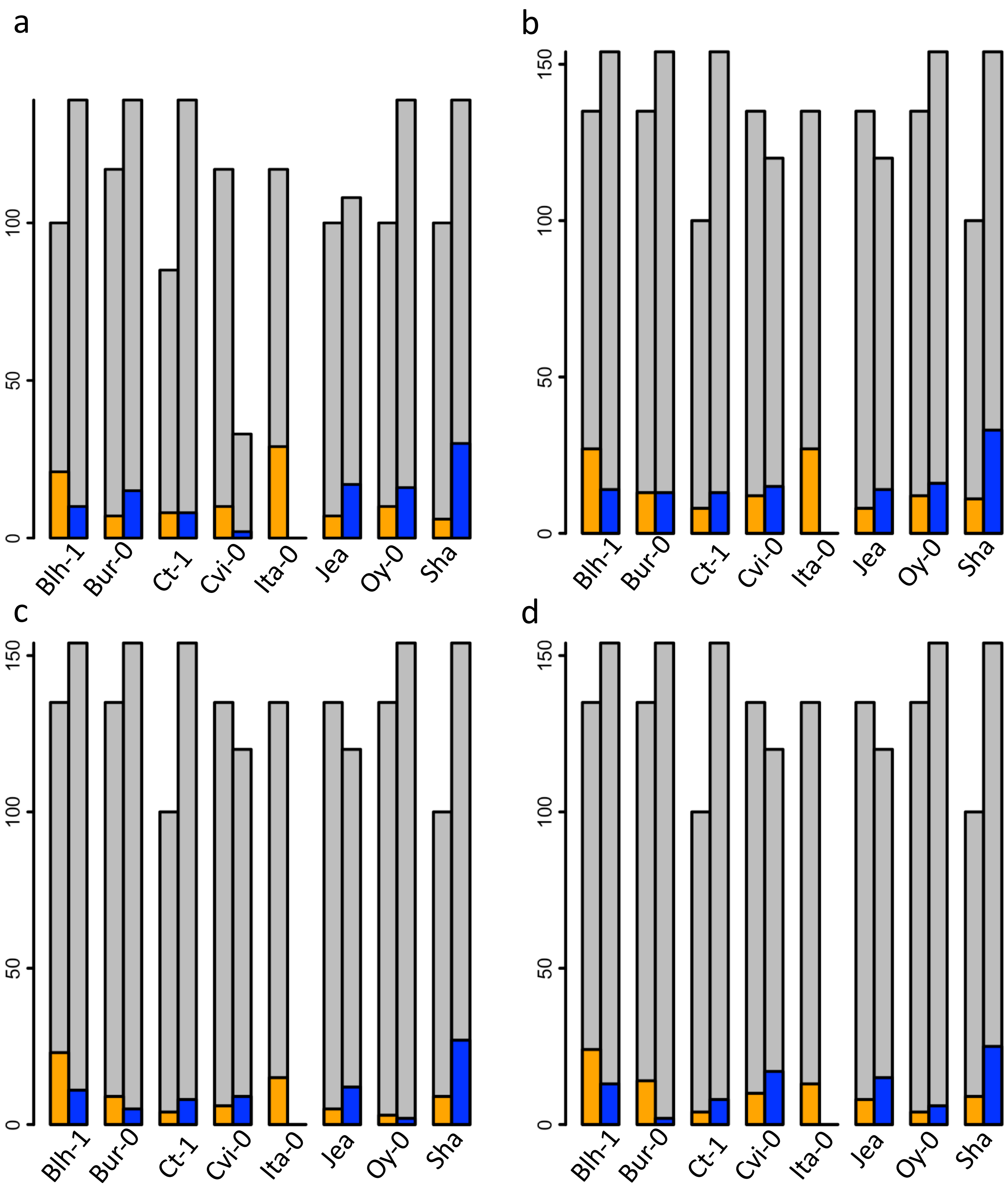

Fig. S4. Contribution of individual accessions to cytonuclear interacting combinations impacting dormancy and germination performance after storage.
The numbers of cytonuclear interacting combinations impacting both germination performance after storage and dormancy as measured at harvest(a), or 3 (b), 6 (c) or 9 (d) months after harvest are plotted according to the contributions of individual accessions. For each accession, the number of significant combinations involving its cytoplasm (orange) or its nucleus (blue) are indicated upon the total number of tested cytonuclear interacting combinations (grey).
