# Supplementary material for: Novel Cytonuclear Combinations Modify Arabidopsis thaliana Seed Physiology and Vigor
Source: Front Plant Sci. 2019 Feb 5;10:32. doi: 10.3389/fpls.2019.00032 (PMC6370702; doi:10.3389/fpls.2019.00032)
Supplement: Supplementary file 13 [file Data_Sheet_5.PDF]

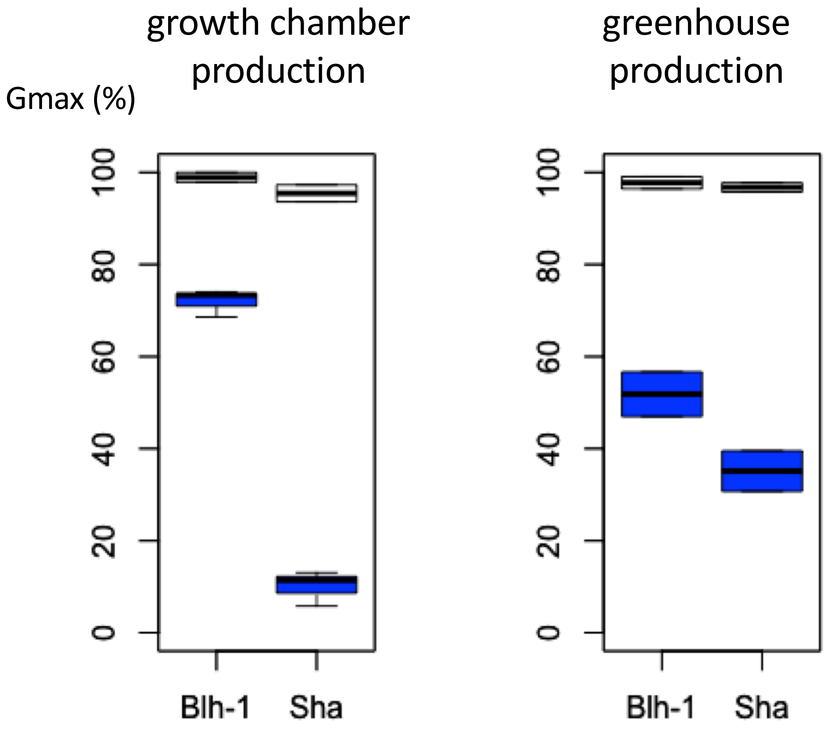

Fig. S5 Germination performance of [Blh-1]Sha and Sha seeds from two environments

[Blh-1]Sha and Sha stratified seeds from growth chamber or greenhouse were tested for germination with and without NaCl (100 mM). The cytoplasmic genome of each genotype is indicated on the x-axis. White boxplots, germination on water; blue boxplots, germination with NaCl. The left panel corresponds to the seed production used in the experiments described in Fig. 2 and Fig. S2.
