# Supplementary material for: Novel Cytonuclear Combinations Modify Arabidopsis thaliana Seed Physiology and Vigor
Source: Front Plant Sci. 2019 Feb 5;10:32. doi: 10.3389/fpls.2019.00032 (PMC6370702; doi:10.3389/fpls.2019.00032)
Supplement: Supplementary file 14 [file Data_Sheet_6.PDF]

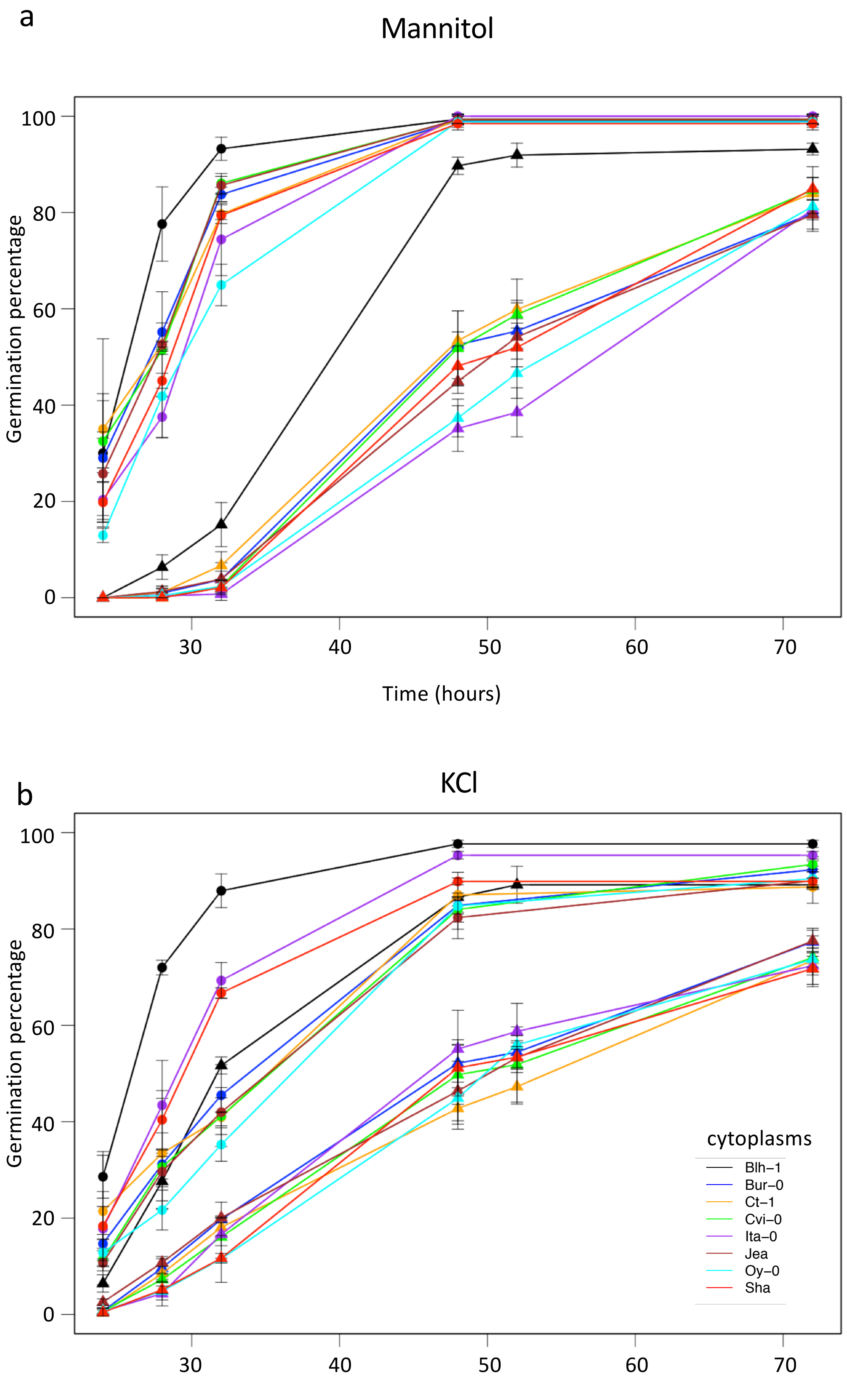

Fig. S6 Germination time curves of the Sha cytoline series on mannitol and KCl.

Germination kinetics of the cytolines, on water (dots) and on (a) 200mM mannitol or (b) 100 mM KCl (triangles). The colors of the graphs indicate the cytoplasms of the genotypes: black, Blh-1; blue, Bur-0; orange, Ct-1; green, Cvi-0; purple, Ita-0; brown, Jea; cyan, Oy-0; red, Sha. Error bars indicate the SD around the plotted mean for germination, from two replicates on water, and three replicates on treatment.
